# Association of trauma, post-traumatic stress disorder and non-affective psychosis across the life course: a nationwide prospective cohort study

**DOI:** 10.1017/S0033291721003287

**Published:** 2023-03

**Authors:** Judith Allardyce, Anna-Clara Hollander, Syed Rahman, Christina Dalman, Stan Zammit

**Affiliations:** 1 Division of Psychological Medicine and Clinical Neuroscience, MRC Centre for Neuropsychiatric Genetics and Genomics, Cardiff University, Cardiff, Wales; 2 Centre for Clinical Brain Sciences (Division of Psychiatry), University of Edinburgh, Edinburgh, Scotland; 3 Dept of Global Public Health, Karolinksa Institutet, Solna, Sweden; 4 Division of Public Health Epidemiology, Department of Public Health Sciences, Karolinska Institutet, Stockholm, Sweden; 5 Psykisk Hälsa, Centrum för epidemiologi och samhällsmedicin, Stockholm, Sweden; 6 Centre for Academic Mental Health, Bristol Medical School, University of Bristol, Bristol, England

**Keywords:** Trauma, psychotic disorders, PTSD, mediation, birth cohort

## Abstract

**Background:**

We aimed to examine the temporal relationships between traumatic events (TE), post-traumatic stress disorder (PTSD) and non-affective psychotic disorders (NAPD).

**Methods:**

A prospective cohort study of 1 965 214 individuals born in Sweden between 1971 and 1990 examining the independent effects of interpersonal and non-interpersonal TE on incidence of PTSD and NAPD using data from linked register data (Psychiatry-Sweden). Mediation analyses tested the hypothesis that PTSD lies on a causal pathway between interpersonal trauma and NAPD.

**Results:**

Increasing doses of interpersonal and non-interpersonal TE were independently associated with increased risk of NAPD [linear-trend incidence rate ratios (IRR)_adjusted_ = 2.17 [95% confidence interval (CI) 2.02–2.33] and IRR_adjusted_ = 1.27 (95% CI 1.23–1.31), respectively]. These attenuated to a relatively small degree in 5-year time-lagged models. A similar pattern of results was observed for PTSD [linear-trend IRR_adjusted_ = 3.43 (95% CI 3.21–3.66) and IRR_adjusted_ = 1.45 (95% CI 1.39–1.50)]. PTSD was associated with increased risk of NAPD [IRR_adjusted_ = 8.06 (95% CI 7.23–8.99)], which was substantially attenuated in 5-year time-lagged analyses [IRR_adjusted_ = 4.62 (95% CI 3.65–5.87)]. There was little evidence that PTSD diagnosis mediated the relationship between interpersonal TE and NAPD [IRR_adjusted_ = 0.92 (percentile CI 0.80–1.07)].

**Conclusion:**

Despite the limitations to causal inference inherent in observational designs, the large effect-sizes observed between trauma, PTSD and NAPD in this study, consistent across sensitivity analyses, suggest that trauma may be a component cause of psychotic disorders. However, PTSD diagnosis might not be a good proxy for the likely complex psychological mechanisms mediating this association.

## Introduction

Psychotic disorders affect approximately 3% of the population at some point in their life (Perälä et al., 2007). This places a substantial burden on patients and healthcare systems. Reducing this morbid and societal burden is further impeded by the limited effectiveness of current treatments, with many sufferers having recurrent episodes, persistent symptoms and/or residual impairment (Owen, Sawa, & Mortensen, 2016).

Extensive evidence for a link between traumatic experiences (TE) and the onset and persistence of psychosis has accumulated over the last decade (Coughlan & Cannon, 2017). Trauma involving interpersonal violence, neglect and intent to harm, appear to be the most salient in the development of psychosis (Arseneault et al., 2011; McGrath et al., 2017a, 2017b; Spauwen, Krabbendam, Lieb, Wittchen, & Van Os, 2018; van Nierop et al., 2014), with the evidence pointing towards the different types or forms of interpersonal trauma having comparable association effect sizes (Croft et al., 2019). Non-interpersonal TE (e.g. loss of a loved one or serious accidental injury) are less studied, but may also be risk factors for psychosis (Abel et al., 2014; McGrath et al., 2017b). Most of the accumulating evidence has focused on childhood trauma with the role of exposure to TE later in the life-course receiving less consideration (Beards et al., 2013; McGrath et al., 2017b). Moreover, exposure to childhood and adulthood trauma are strongly correlated (Stain et al., 2013), with TE tending to co-occur or cluster within individuals (Green et al., 2010; Kessler et al., 2010; McGrath et al., 2017a) and possibly exerting a cumulative effect, related to both the number and type of trauma (Arseneault et al., 2011; Croft et al., 2019; Janssen et al., 2004; Kelleher et al., 2013).

History of traumatic or stressful life events are diagnostic criteria for post-traumatic stress disorder (PTSD) and acute stress reaction (ASR), and while research has mainly focused on PTSD, both have a high rate of comorbidity with psychotic disorders (Buckley, Miller, Lehrer, & Castle, 2008). Recent prospective studies have further shown that having a diagnosis of PTSD or ASR is associated with both an increased risk of psychotic disorders (Okkels, Trabjerg, Arendt, & Pedersen, 2016) and psychotic experiences (Lewis et al., 2019).

It has been hypothesised that PTSD mediates the association between trauma and psychosis severity and functional impairment, for at least a proportion of individuals (Mueser, Rosenberg, Goodman, & Trumbetta, 2002; Williams, Bucci, Berry, & Varese, 2018). The development of such explanatory models are needed to extend our understanding and explain the interrelationship between trauma, PTSD and psychotic disorder in order to inform the development of prevention strategies and novel trauma-informed interventions for psychosis (Brand, McEnery, Rossell, Bendall, & Thomas, 2018). Whilst there is some evidence to support this hypothesis, there are several areas where the evidence is weak or missing and we attempt to address these here.

First, although longitudinal studies show consistent evidence of an association between traumatic and psychotic experiences, there are few studies of psychotic disorders. While psychotic experiences in individuals with and without clinically diagnosed psychotic disorders are phenomenologically similar, there are large differences in their associated emotional valence, distress and functional impairment (Daalman et al., 2012; Lawrie, Hall, McIntosh, Owens, & Johnstone, 2010) and possibly their aetiology. For example, paranoid ideation is a common consequence of abuse, but these persecutory thoughts may have a different aetiology to the persecutory experiences and delusions which characterise psychotic disorders. Furthermore, psychotic experiences may be better conceptualised as markers of general psychopathology, rather than as specific risk factors for psychotic disorders such as schizophrenia (Fisher et al., 2013; Stochl et al., 2015).

While studies of psychotic experiences show an effect gradient and stronger associations with interpersonal than non-interpersonal trauma, there are currently no longitudinal studies which examine dose-response relationships or compare the effects of non-interpersonal and interpersonal TE on the incidence of psychotic disorders. Studies of psychotic disorder have almost all used cross-sectional data (Varese et al., 2012), which hampers causal inference due to difficulties untangling causal effects from reverse causation and residual confounding in retrospective (post-outcome) assessment of exposures.

Second, although PTSD is common in people with a psychotic disorder (Seow et al., 2016), only two studies have examined whether people with PTSD have an increased risk of subsequently developing a psychotic disorder, only one of which examined the extent to which PTSD symptoms mediate the association between trauma and psychotic disorder (although these were not clinician diagnoses) (Strelchuk et al., 2020). A few studies have examined whether PTSD symptoms mediate the relationship between trauma and psychotic experiences rather than psychotic disorder (Williams et al., 2018), but apart from one (Strelchuk et al., 2020), the rest have all have been cross-sectional in design and unable to determine direction of effect.

To date, no longitudinal studies have examined exposure to trauma, PTSD, and psychotic disorder in a unitary sample to address these limitations. Therefore, we use data from the Swedish national register to examine whether: (i) interpersonal and non-interpersonal traumatic events (TE) are associated with subsequent non-affective psychotic disorders (NAPD), (ii) PTSD is associated with subsequent NAPD, and (iii) the extent to which the association between TE and NAPD is mediated by PTSD.

## Methods

### Study cohort

The data used in this study was extracted from Psychiatry Sweden (PS) – a comprehensive linked national record register, which was developed specifically for mental health research. Record linkage brings together two or more records relating to the same individual so that information from multiple sources can be joined to produce richer data for research purposes in public health and epidemiological research. Record linkage matches a person' records via a unique identifier. Sweden has a strong track record for performing record linkage for research purposes owing to its routinely collected and well-maintained national administrative health data sets.

We identified all individuals born in Sweden between 1 January 1971 and 31 December 1990, who were resident in Sweden on their 16th birthday from the total population register (RTP) (*N* = 1 965 214). This cohort was then followed up from their 16th birthday until they either received a first-ever diagnosis of the outcome of interest, censorship due to death, emigration, or the study end-date (31 December 2016), whichever came first.

The Swedish National register diagnoses are well recognised as having good validity for psychiatric epidemiological studies (Ludvigsson et al., 2011). As PTSD has high co-morbidity with other psychiatric disorders, further validation studies have been carried out which show its reliability and validity is adequate for large scale epidemiological studies in the Swedish population during the time period of these analyses (Hollander et al., 2019).

### Assessment of NAPD and PTSD

The cohort was linked to Sweden' National Patient Registers which used International Statistical Classification of Diseases and Related Health Problems, Ninth Revision (World Health, 1978) for the period 1st January 1987–31st December 1996, and Tenth Revision (World Health, 2004) from 1 January 1997 onwards. Data for NAPD (coded as ICD-9: 295, 297, 298.2–298.9; ICD-10: F20–F29) is reliable, with complete inpatient coverage for the whole study period. Inpatient admissions are reliably coded for PTSD (ICD-10: F43.1) from 1 January 1997. Complete outpatient coverage for NAPD and PTSD were available from 1 January 2006. We also examined acute stress reaction (ASR: ICD-9: 308, ICD-10: F43.0) in a sensitivity analysis (see below).

### Trauma exposure

Probable TE was identified using ‘reason for contact’ coding extracted from the national patient register. We derived two pre-specified categories of exposure for TE which are used extensively in trauma research: non-interpersonal and interpersonal TE (Aas et al., 2013). Non-interpersonal TE was defined as the presence of either (i) death of a first degree relative (mother, father, full sibling, child) or of a partner/spouse, or (ii) injury due to accidents without violence (for further details and ICD codes, see online Supplementary S Notes 1: Definition using ICD codes for Traumatic Experiences). For the former, relatives were identified from the Multigenerational Register and linked to the Cause of death Register to extract the date of deaths of first-degree relatives. Partners were defined as husbands, wives, common law spouses, registered partners, or another adult living in the same household as the proband, ascertained from the quinquennial Population and Housing Censuses of 1985 and 1990 and from the annual LISA (Longitudinal Integration Database for Health Insurance and Labour Market Studies) returns thereafter. Records from any identified partner were linked to the Cause of Death Register with their death included as an exposure if it occurred during the period of shared residency. Interpersonal TE were defined as injury due to war, terrorism, iatrogenic/medical misadventure, and violence/assaults (further details and ICD codes: online Supplementary S Notes 1: Definition using ICD codes for TE).

### Covariates

Age (5-year age-bands), calendar-period (5-year bands) and sex were obtained from the RTP. Paternal age at off-spring' conception was dichotomised at > age 39, guided by recent meta-analyses supporting a cut off between 30 and 39 years of age (Miller et al., 2011). Second-generation migrant status was extracted from Multi-Generational Registers linked to RTP/STATIV. Compulsory school best educational attainment was extracted from LISA, and parents' socioeconomic status at participant' birth from quinquennial censuses (1970–1990). Parental lifetime history of severe mental illness (SMI) was indexed in three separate binary variables as a proxy for familial liability to: (i) NAPD (as above), (ii) mania (296.1–296.3, F30–31) and (iii) depression (296.2, 300.4, F32–39).

### Statistical analyses

Data were analysed using STATA 15 (StataCorp, 2017). Piecewise exponentiated survival models were fitted within the generalised linear modelling framework using log-linear Poisson Regression (Royston, 2011). Age, calendar-period, non-interpersonal and interpersonal TE were all considered as time-dependent variables (Clayton & Hills, 2013). The numbers of interpersonal and non-interpersonal TE before the age of 16 were entered at baseline. All TEs after age 16 were handled as time-varying covariates entering the model at the time they were first coded in the health service records. Using episode splitting the person-time exposure load was partitioned more precisely for each level (count) of exposure (for more information see online Supplementary S Note 2: Methods: exponentiated survival models and time-dependent variables). Sex, paternal age, compulsory school educational attainment, second-generation migrant status, socioeconomic status, and parental lifetime history of SMI were all entered as time-fixed covariates.

We examined the effect of interpersonal and non-interpersonal TE as they accrued, until failure (time when a person either experienced the outcome of interest or was censored), on the first-ever diagnosis of NAPD and PTSD, estimating incidence rate ratios (IRRs) in separate time to first-event analyses. The initial forced models included age, sex (and their interaction) and calendar period. We extended this model controlling for each confounder in turn and finally included all the covariates, with the fully adjusted model including mutual adjustment for the alternative type of traumatic event. A complementary set of analyses using the same statistical approach was used to examine the effect of PTSD on risk of NAPD. TE were modelled both as indicator variables to estimate category-specific estimates, and as a continuous exposure to estimate a linear-trend estimate across categories.

To evaluate the robustness of our findings we conducted a series of sensitivity analyses: (1) To assess possible reverse causation due to undetected prodromal psychosis which may increase exposure to TE (Carmen, Rieker, & Mills, 1984), we imposed induction lengths of 1–5 years between date of TE and first-ever diagnosis of NAPD and estimated lagged time-dependent IRRs; (2) To examine whether any observed association between TE and first-ever diagnosis of NAPD was driven by factors associated with childhood trauma, we restricted analyses to TE occurring after age 16, and subsequently adjusted for recorded childhood trauma; (3) To address possible misclassification of prodromal psychosis as PTSD, we performed a series of analyses imposing induction lengths of 1–5 years between first-ever diagnosis of PTSD and first-ever diagnosis of NAPD; (4) To evaluate whether the association of PTSD with NAPD was stronger (more specific) than for other stress-related disorders, we repeated these analyses using ASR instead of PTSD.

Finally, mediation analysis within a counterfactual framework (Robins & Greenland, 1992) was performed using the inverse odds weighting (IOW) method (Hossin, Koupil, & Falkstedt, 2019; Nguyen, Osypuk, Schmidt, Glymour, & Tchetgen Tchetgen, 2015; Tchetgen Tchetgen, 2013; VanderWeele & Vansteelandt, 2014) to estimate natural direct and indirect effects (Pearl, 2000) when PTSD is included on the pathway from interpersonal TE to first-ever NAPD. We chose, *a priori*, to examine interpersonal TE in the mediation model as they have been consistently shown to be more strongly associated with both PTSD (Kessler et al., 2017) and NAPD (Arseneault et al., 2011). Interpersonal TE was dichotomised to optimise the effectiveness of this weighted approach (Nguyen et al., 2015; VanderWeele & Vansteelandt, 2014) and entered as a time-dependent variable at the time of the first trauma-related code in the health register. Standard errors were bootstrapped (500 replications) and percentile-based confidence intervals (CIs) derived. Models were adjusted for the full battery of confounders described above.

## Results

### Cohort characteristics

1 873 593 individuals with no missing data (>95% original cohort) were included in the analyses (baseline characteristics reported in online Supplementary Table S1: Cohort characteristics by non-affective psychosis status). From 1987–2016 (36 290 751 person-years at-risk) 17 653 individuals (0.94%) had a first-ever diagnosis of NAPD and 11 225 (0.6%) had a first-ever diagnosis of PTSD. Non-interpersonal TE was commoner than interpersonal TE, with 299 569 persons (16%) exposed to 1 or more non-interpersonal TE across the follow-up period compared to 14 528 (0.78%) exposed to interpersonal TE, consistent with other register-based studies (Webb et al., 2017).

### Relative risk for NAPD by background TE exposure

**Fig. 1.**
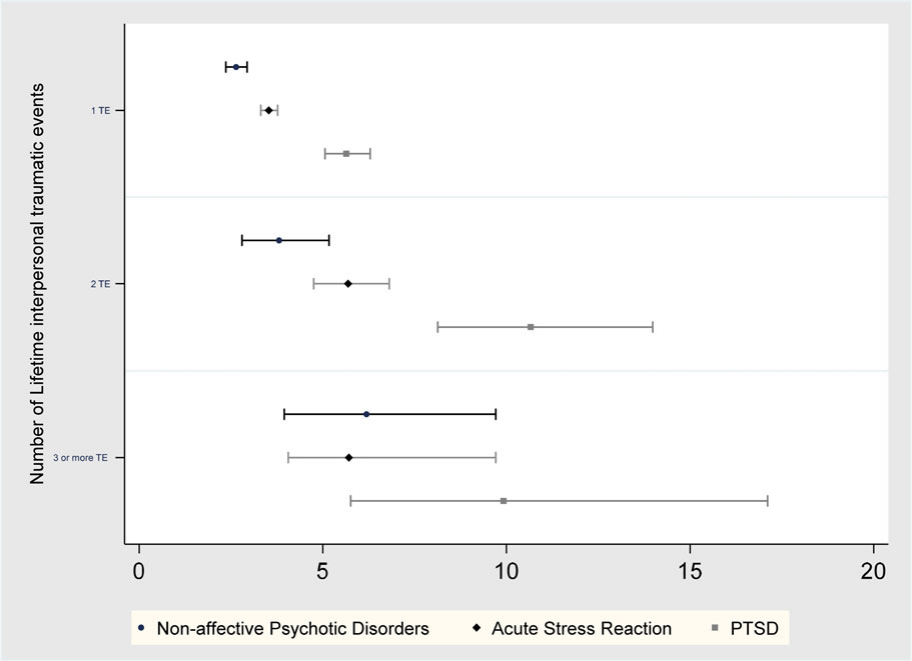
Coefficient plot: IRR for psychiatric outcomes according to number of interpersonal TE across life-course. IRRs are estimated adjusted for age, sex (and their interaction), calendar period, paternal lifetime history of NAPD, depressive disorders and bipolar-disorder, socioeconomic status, paternal, age at conception, compulsory school education attainment, second-generation migrant status for age, sex, period, paternal age, second-generation migrant status, paternal history of depressive disorder, NAPD and BD, socioeconomic status and best school educational attainment, NAPD, non affective psychotic disorders, ASR acute stress reaction, PTSD post-traumatic stress disorder.

Non-interpersonal and interpersonal TE were independently associated with risk of NAPD, which persisted after adjustment for confounders (Table 1, section A, Fig. 1). IRRs for NAPD stratified by interpersonal TE exposure were substantially larger than for non-interpersonal TE, with non-overlapping CIs. There was a monotonic increase in IRRs with increasing number of interpersonal TE.

**Table 1.** Association between TE and first-ever diagnosis of NAPD and PTSD

TE	Model 1 (forced) Adjusted for calendar year age, sex and age × sex interaction				Model 2 (full) Adjusted forced + complement of confounder variables ^a^			
A). Outcome: Non affective Psychotic Disorders (NAPD)								
Number of Non-interpersonal TE								
	IRR	*p* Value	95% CI		IRR	*p* Value	95% CI	
1	1.57	< 0.0001	1.50	1.64	1.27	<0.0001	1.21	1.32
2	2.52	<0.0001	2.26	2.81	1.74	<0.0001	1.54	1.93
3 or more	2.54	<0.0001	1.90	3.38	1.70	<0.0001	1.27	2.26
Rate ratio linear trend	1.54	<0.0001	1.49	1.59	1.27	<0.0001	1.23	1.31
# Interpersonal TE								
1	4.14	<0.0001	3.71	4.61	2.64	<0.0001	2.36	2.94
2	6.84	<0.0001	5.03	9.30	3.81	<0.0001	2.80	5.17
3 or more	11.68	<0.0001	7.45	18.33	6.19	<0.0001	3.95	9.71
Rate ratio linear trend	2.95	<0.0001	2.76	3.16	2.17	<0.0001	2.02	2.33
B). Outcome: PTSD								
Number of Non-interpersonal TE								
1	1.72	< 0.0001	1.63	1.81	1.43	<0.0001	1.36	1.51
2	3.04	<0.0001	2.69	3.42	2.20	<0.0001	1.94	2.47
3 or more	5.03	<0.0001	3.87	6.54	3.41	<0.0001	2.62	4.44
Rate ratio linear trend	1.71	<0.0001	1.65	1.77	1.45	<0.0001	1.39	1.50
# Interpersonal TE								
1	9.22	<0.0001	8.28	10.27	5.64	<0.0001	5.06	6.29
2	21.09	<0.0001	16.08	27.64	10.66	<0.0001	8.13	13.98
3 or more	19.50	<0.0001	11.31	33.60	9.92	<0.0001	5.76	17.11
Rate ratio linear trend	4.73	<0.0001	4.45	5.03	3.43	<0.0001	3.21	3.66

a Full Model: adjusted for age, sex (and their interaction), calendar period, paternal lifetime history of NAPD, depressive disorders and bipolar disorder, socioeconomic status, paternal. age at conception, compulsory school education attainment, second-generation migrant status. This model is mutually adjusted for interpersonal and non-interpersonal TEs.

### Relative risk for PTSD by background TE exposure

Interpersonal and non-interpersonal TE were independently associated with increased risk for PTSD (Table 1, section B). Risk was substantially higher for interpersonal than non-interpersonal TE after mutual adjustment and after controlling for confounding.

### PTSD and risk of NAPD

PTSD was associated with an approximately 15-fold increased risk for NAPD (Table 2). The effect-estimate was attenuated after adjusting for covariates, although there remained strong evidence of increased risk of NAPD after adjustment for all measured confounding variables [lRR = 8.06 (95% CI 7.23–8.99)].

**Table 2.** Associations between PTSD/ASR and first-ever diagnosis of NAPD

Diagnostic Group	Model 1 (forced) adjusted for calendar year, age, sex and age × sex interaction				Model 2 (Full)^b^ Adjusted as forced model1 + full complement of confounder variables ^b^			
	IRR	*p* Value	95% CI		IRR	*p* Value	95% CI	
a.								
PTSD	14.83	<0.0001	13.31	16.53	8.06	<0.0001	7.23	8.99
Lagged 1 YEAR					6.91	<0.0001	6.08	7.85
Lagged 2 YEAR					6.17	<0.0001	5.32	7.15
Lagged 3 YEAR					5.46	<0.0001	4.58	6.49
Lagged 4 YEAR					5.20	<0.0001	4.26	6.35
Lagged 5 YEAR					4.62	<0.0001	3.65	5.87
b.								
ASR	9.02	<0.0001	8.44	9.64	5.63	<0.0001	5.27	6.02
Lagged 1 YEAR					4.50	<0.0001	4.11	4.81
Lagged 2 YEAR					4.07	<0.0001	3.73	4.45
Lagged 3 YEAR					3.87	<0.0001	3.50	4.26
Lagged 4 YEAR					3.79	<0.0001	3.39	4.22
Lagged 5 YEAR					3.68	<0.0001	3.26	4.16

Full model: adjusted for age, sex (and their interaction), calendar period, paternal lifetime history of NAPD, depressive disorders and bipolar-disorder, socioeconomic status, paternal. age at conception, compulsory school education attainment, second-generation migrant status.

### Mediation analyses

There was little evidence that PTSD mediated the association between interpersonal TE and NAPD (natural indirect (mediation) effect: IRR = 0.92; percentile CI 0.80–1.07; Table 3).

**Table 3.** Mediation of the association between interpersonal TE and NAPD by PTSD

	IRR	Bootstrap s.e.	Percentile CI
Total effect^a^	2.42	0.05	2.18–2.66
Natural direct effect^b^	2.62	0.09	2.18–3.10
Natural indirect effect	0.92	0.07	0.80–1.07

a Adjusted for age, sex (and their interaction), calendar period, paternal lifetime history of NAPD, depressive disorders and bipolar-disorder, socioeconomic status, paternal. age at conception, compulsory school education attainment, second-generation migrant status.

b Obtained by applying the inverse odds weights with full complement of adjustments described.

### Sensitivity analyses

(1) Imposing induction lengths of 1–5 years between TE exposure and first ever NAPD to assess the role of reverse causation, IRRs remained substantively unchanged, with only a relatively small attenuation in 5-year lagged time-dependent IRRs for both non-interpersonal and interpersonal traumas (online Supplementary Table S2). (2) The association between both interpersonal and non-interpersonal TE and NAPD persisted when trauma exposure was restricted to traumas experienced after age 16, and after adjusting for childhood TE (online Supplementary Table S3). (3) 5-year lagged time-dependent IRRs for NAPD after a diagnosis of PTSD (Table 2) were attenuated by approximately 50%, although remained substantial (lagged IRR_5yr_ 4.62, 95% CI 3.65–5.87). (4) ASR associations with both TE and NAPD were weaker than for PTSD, though results were substantively similar (online Supplementary Table S4).

## Discussion

### Trauma and NAPD

Using data from a Swedish nationwide birth cohort we show clear temporal relationships between exposure to trauma and subsequent risk of NAPD. Our analyses account for both type and number of TE, in a time-to-event (survival) framework. We adjust for the alternative trauma-type to try and disentangle non-interpersonal and interpersonal trauma effects, which tend to co-occur (Kessler et al., 2018), and find that non-interpersonal and interpersonal traumas are independently associated with increased NAPD risk. The specific effect for interpersonal trauma is substantially stronger however, and shows a clearer effect gradient, consistent with published reports of higher rates of psychotic experiences in adolescents exposed to trauma involving intention to harm, threat and hostility (Arseneault et al., 2011). While non-interpersonal trauma is less studied, the small/modest independent increase in risk of NAPD observed is consistent with other (Abel et al., 2014; Arseneault et al., 2011), although not all (Moriyama et al., 2018) studies.

Evidence of association between trauma and risk of NAPD remained strong after adjustment for a range of measured confounders, but estimates were attenuated, particularly for interpersonal trauma, suggesting these association are more prone to residual confounding. Individuals who develop NAPD have (on average) higher rates of subtle cognitive and neurodevelopmental abnormalities which may share genetic liability with NAPD and influence exposure to trauma. While parental history of psychiatric disorders will not capture all relevant shared genetic risk, our estimates for interpersonal trauma remained large and almost unchanged after adjustment for family history, making it less likely that observed associations are substantially attributable to genetic confounding. Systematic reviews of family and twin studies suggest genetic effects have moderate influences on environmental exposures (Kendler & Baker, 2007), though adjusting for schizophrenia-related polygenic risk did not attenuate associations between trauma and psychotic experiences in another cohort study (Croft et al., 2019).

Our results were substantively unchanged when we examined trauma exposure in adulthood and adjusted for childhood trauma, consistent with the allostatic load hypothesis which suggests it is trauma accumulation across the life-course which increases psychosis risk.

### Trauma, PTSD and NAPD

Whilst we replicate findings from a Danish study showing that traumatic stress disorders are associated with increased risk of subsequent NAPD (Okkels et al., 2016), we extend this by showing a stronger effect for PTSD than ASR and by examining the extent to which PTSD mediates the association between trauma and psychosis risk. Despite strong associations between interpersonal trauma and both PTSD and NAPD, and between PTSD and subsequent NAPD, we found little support for the hypothesis that PTSD mediates the relationship between trauma and psychosis. However, people with PTSD do not always present to secondary care (Lewis et al., 2019) and where they do they are likely to receive treatment for their PTSD symptoms; therefore our results may under-estimate the mediation effect of PTSD symptoms.

This study provides novel information that can help inform our understanding of how TE can lead to the development of psychosis. The absence of evidence for PTSD mediation in our study suggests that the conceptualisation of trauma leading to PTSD symptoms which, over time, develop into psychosis might need reframing, although are findings are not necessarily inconsistent with the hypothesis that traumatic pathology has a causal role in the development of psychosis. The potentially causal relationship between trauma and psychotic disorders are likely to be complex and a measure of PTSD diagnosis may not be the best proxy for the multiple pathways and psychological mechanisms underpinning this association. Examination of potentially specific trauma-symptom associations (Hardy, 2017) or of specific types of TE such as sexual abuse which we have not measured in this study, might provide a more sophisticated understanding of this relationship.

An alternative hypothesis suggests there is a spectrum of multifaceted, dimensional psychopathological responses to trauma, ranging in severity and need for care, and it is the balance between the timing, severity, and repetitiveness of trauma exposure within the context of an individual' specific liability (genetic, psychological and social) which leads to differential psychopathological expressions of response to trauma (Morrison, Frame, & Larkin, 2003). That is, while ASR, PTSD, and NAPD diagnostic categories are clinically useful, they may well be artificial constructs lying along trauma-related phenomenological continua rather than representing discrete/separate psychopathological processes.

### Strengths and limitations

This study used data from a large national cohort, representative of the Swedish population and therefore is less likely to be affected by selection bias. TE were recorded prospectively and independently of outcome using contemporaneous register data, hence eliminating recall bias. Furthermore, we used a Poisson framework in our analyses so that time could be considered a covariate rather than an outcome, to adjust for potential systematic variation in estimated rates due to secular changes in diagnostic approaches and other period effects. We also conducted a series of sensitivity analyses to test the robustness of our findings. For example, although the average psychosis prodrome is approximately 2 years (Loebel et al., 1992), we observe a relatively small attenuation of estimates in the 5-year lagged models, making it unlikely reverse causation explains the associations we observe.

However, our results should be interpreted in the context of several limitations. First, although we adjust for a range of administratively available covariates in the analyses, some residual confounding is probable, and a well-recognised issue in all register-based studies (Mortensen, Allebeck, & Munk-Jørgensen, 1996). There might also be residual confounding for some of the measures we adjust for, such as socio-economic status and educational attainment as these are relatively lacking in detail. Given that both are more strongly associated with inter-personal than non-interpersonal trauma (Breslau et al., 1998), there might be more scope for residual confounding affecting estimates for inter-personal trauma in our study.

Second, as we use administrative data originally collected for health service purposes (such as providing valid health data and statistical information to support health service development) to measure trauma, our estimates reflect effects of trauma requiring hospital contact. This approach will have underestimated exposure to all the traumas that would meet criteria for trauma as defined by DSM or ICD diagnostic manuals, missing events such as childhood abuse or neglect, and witnessing or learning of TE as they occurred in others, as well as other traumas that would not have led to a hospital contact.

Furthermore, interpersonal TE may be misclassified as accidents where intent is undetermined/unknown (Ballard, Kalb, Vasa, Goldstein, & Wilcox, 2015), and whilst we coded medical misadventures as interpersonal traumas it could equally be argued that these are non-interpersonal, though the number of individuals coded as such was small. Therefore, while the hospital contact codes we have used in this study are likely to indicate a level of trauma that is clinically relevant, our estimates might not extrapolate well to all traumas, and be affected by misclassification bias. If non-differential, this would likely lead to an under-estimate of effects. However, if propensity for hospital contact following a trauma differed between those who eventually developed PTSD or NAPD compared to those who did not, this could have led to either over- or under-estimation of effects.

Similar issues apply to our outcome measures as we were unable to identify individuals in the population with symptoms of PTSD or NAPD who either received no treatment or only received treatment in primary or private care. However, using clinically determined diagnoses makes these results readily generalisable to clinical settings, even if not to the whole population. We were unable to examine complex PTSD that is perhaps a more pertinent mediator for the effects of trauma on psychosis as this diagnosis has only been introduced in ICD-11 (Cloitre, Garvert, Brewin, Bryant, & Maercker, 2013).

Third, PTSD and NAPD have high levels of comorbidity (Achim et al., 2009) and their psychopathology substantially overlap (Alsawy, Wood, Taylor, & Morrison, 2015; Scott, Nurcombe, Sheridan, & McFarland, 2007). For example, hallucinations in NAPD are analogous to flashbacks in PTSD, suspicion resembles hypervigilance and negative symptoms overlap with the numbing and avoidance seen in PTSD, which may make it difficult to differentiate PTSD and NAPD phenomenologically (Jessop, Scott, & Nurcombe, 2008). If this diagnostic uncertainty or comorbidity is coded or handled differently across individual clinicians and hospital sites, it may reduce inter-diagnostic discrimination and reliability and result in misclassification of PTSD, which is already recognised to be under-detected in secondary care settings (Zammit et al., 2018). Fourth, our analytical approach required all cohort members to have been born in Sweden and survive to 16 years, with trauma exposure in childhood entered at baseline to prevent immortal time bias (time-dependent bias); however, this method precludes the examination of critical or sensitive periods of trauma exposure during childhood. Finally, we used dichotomised measures in the mediation analysis to optimise the IOW approach (Nguyen et al., 2015; VanderWeele & Vansteelandt, 2014) but this will have resulted in information loss regarding exposure dosage, and similarly to the absence of information on the provision or success of any treatment provided for PTSD, could potentially have led to underestimation of the mediation effect in our study.

### Implications

The large effect sizes observed when examining the associations between TE, PTSD and NAPD in this study, and our study design and sensitivity analyses to minimise effects of confounding, reverse causation and bias (though see limitations above) is consistent with trauma being a component cause in the onset of psychotic disorders. However, our mediation results suggest that a diagnostic category of PTSD is unlikely to substantially mediate this relationship. It is unclear therefore whether PTSD symptoms lie on the causal pathway to psychosis, or whether PTSD and psychosis lie on a spectrum of trauma-related psychopathological expression. A clearer conceptualisation of how psychosis can develop following trauma is likely to provide novel opportunities for improving treatment outcomes in psychotic disorders (Alameda et al., 2020; Compean & Hamner, 2019).

## Supplementary material

For supplementary material accompanying this paper visit https://doi.org/10.1017/S0033291721003287. click here to view supplementary material
