# Supplementary material for: Association of trauma, post-traumatic stress disorder and non-affective psychosis across the life course: a nationwide prospective cohort study
Source: Psychol Med. 2021 Aug 20;53(4):1620–8. doi: 10.1017/S0033291721003287 (PMC10009379; doi:10.1017/S0033291721003287)
Supplement: Supplementary file 1 [file S0033291721003287sup001.docx]

**Supplementary Notes**

**S1**

The trauma measures used in these analyses were the ‘‘reason for contact’’ coding recorded in the national patient register from the list presented below. As we are using routinely collected data we have no measure of subjective experience of the traumatic event. Non-interpersonal TE was defined as the presence of either i) death of a first degree relative (mother, father, full sibling, child) or of a partner/spouse. Relatives were identified from the Multigenerational Register and linked to the Cause of death Register to extract the deaths of first-degree relatives. Partners were defined as husbands, wives, common law spouses, registered partners or another adult living in the same household as the proband, ascertained from the quinquennial Population and Housing Censuses of 1985 and 1990 and from the annual LISA returns (Longitudinal Integration Database for Health Insurance and Labour Market Studies) thereafter. Records from any identified partner were linked to the Cause of Death Register with their death included as an exposure if it occurred during the period of shared residency. ii) Injury due to accidents without violence. Interpersonal TE were defined as injury due to war, terrorism, iatrogenic/medical misadventure and violence/assaults.

**Definition using ICD codes for Traumatic Experiences.**

**Nonviolent accidents**

*-----------------ICD 9

E800-E807 Railway Accidents

E810-E819 Motor Vehicle Traffic Accidents

E820-E825 Motor Vehicle Nontraffic Accidents

E826-E829 Other Road Vehicle Accidents

E830-E838 Water Transport Accidents

E840-E845 Air and Space Transport Accidents

E846-E849 Vehicle Accidents, Not Elsewhere Classifiable

E880-E888 Accidental Falls

E890-E899 Accidents Caused by Fire and Flames

E900-E909 Accidents Due to Natural and Environmental Factors

E910-E915 Accidents Caused by Submersion, Suffocation, And Foreign Bodies

E916-E928 Other Accidents

*----------------ICD 10

V00-V09 Pedestrian injured in transport accident.

V10-V19 Pedal cycle rider injured in transport accident.

V20-V29 Motorcycle rider injured in transport accident.

V30-V39 Occupant of three-wheeled motor vehicle injured in transport accident.

V40-V49 Car occupant injured in transport accident.

V50-V59 Occupant of pick-up truck or van injured in transport accident.

V60-V69 Occupant of heavy transport vehicle injured in transport accident.

V70-V79 Bus occupant injured in transport accident.

V80-V89 Other land transport accidents

V90-V94 Water transport accidents

V95-V97 Air and space transport accidents

V98-V99 Other and unspecified transport accidents

W00-W19 Slipping, tripping, stumbling and falls.

W20-W49 Exposure to inanimate mechanical forces

W50-W64 Exposure to animate mechanical forces.

W65-W74 Accidental non-transport drowning and submersion

W85-W99 Exposure to electric current, radiation and extreme air temperature or pressure

X00-X08 Exposure to smoke, fire and flames

X10-X19 Contact with heat and hot substances.

X30-X39 Exposure to forces of nature

X50-X50 Overexertion and strenuous or repetitive movements

X52-X58 Accidental exposure to other specified factors

**War, Terrorism, medical misadventure, violence, and assaults**

ICD 9

E960-E969 & E979 & E999.1

ICD 10

X85-Y09 & Y87.1 - *U01-*U02

**Homicide and Injury Purposely Inflicted by Other Persons**

*-------------------ICD 9

E960 Fight brawl rape

E961 Assault by corrosive or caustic substance, except poisoning

E962 Assault by poisoning

E963 Assault by hanging and strangulation.

E964 Assault by submersion [drowning]

E965 Assault by firearms and explosives

E966 Assault by cutting and piercing instrument

E967 Perpetrator of child and adult abuse

E968 Assault by other and unspecified means

E969 Late effects of injury purposely inflicted by other person.

E979 Terrorism

E999.1 Terrorism

*---------------------ICD 10

X85 Assault by drugs, medicaments, and biological substances

X86 Assault by corrosive substance

X88 Assault by gases and vapours

X89 Assault by other specified chemicals and noxious substances

X90 Assault by unspecified chemical or noxious substance

X91 Assault by hanging, strangulation and suffocation.

X92 Assault by drowning and submersion.

X93 Assault by handgun discharge

X94 Assault by rifle, shotgun and larger firearm discharge

X95 Assault by other and unspecified firearm discharge

X96 Assault by explosive material

X97 Assault by smoke, fire and flames

X98 Assault by steam, hot vapours and hot objects

X99 Assault by sharp object

Y00 Assault by blunt object

Y01 Assault by pushing from high place.

Y02 Assault by pushing or placing victim before moving object.

Y03 Assault by crashing of motor vehicle.

Y04 Assault by bodily force

Y05 Sexual assault by bodily force

Y06 Neglect and abandonment

Y07 Other maltreatment

Y08 Assault by other specified means

Y09 Assault by unspecified means

Legal Wars & other legal interventions

*------------ICD 9

E970 Injury due to legal intervention by firearms

E971 Injury due to legal intervention by explosives

E972 Injury due to legal intervention by gas

E973 Injury due to legal intervention by blunt object

E974 Injury due to legal intervention by cutting and piercing instrument

E975 Injury due to legal intervention by other specified means

E976 Injury due to legal intervention by unspecified means

E977 Late effects of injuries due to legal intervention

E978 Legal execution

War

E990 Injury due to war operations by fires and conflagrations

E991 Injury due to war operations by bullets and fragments

E992 Injury due to war operations by explosion of marine weapons

E993 Injury due to war operations by another explosion

E994 Injury due to war operations by destruction of aircraft

E995 Injury due to war operations by unspecified forms -conventional warfare

E996 Injury due to war operations by nuclear weapons

E997 Injury due to war operations by other forms of unconventional warfare

E998 Injury due to war operations but occurring after ceasing hostilities.

E999 Late effect of injury due to war operations and terrorism

-----------------------ICD 10

Y35 Legal intervention

Y36 Operations of war

Y37 Military operations

Y38 Terrorism

Misadventures to Patients During Surgical and Medical Care

*--------ICD 9

E870 Accidental cut puncture perforation or haemorrhage during medical care

E871 Foreign object left in body during procedure.

E872 Failure of sterile precautions during procedure

E873 Failure in dosage

E874 Mechanical failure of instrument or apparatus during procedure

E875 Contaminated or infected blood other fluid drug or biological substance

E876 Other and unspecified misadventures during medical care

[Surgical and Medical Procedures as The Cause Of Abnormal Reaction Of Patient

Or Later Complication, Without Mention of Misadventure at The Time

Of Procedure E878-E879]

E878 Surgical operation and other surgical procedures as the cause

E879 Other procedures without mention of misadventure at the time

*---------icd 10

Y40 - y84

Note on Medical Misadventure

It is unclear where medical misadventure should be included as an interpersonal or non-interpersonal trauma. There is increasing literature regarding post-hospitalisation trauma – in which the trauma is at least in part a consequence of medical treatment (including, for example, being on ICU, even where everything went as planned). However, here we only used codes related to ‘medical misadventure’ e.g., foreign bodies left after surgery, and overdoses or accidental exposure to radiation. Following discussion, we opted to classify these as inter-personal traumas as they were caused by errors or neglect within the context of a health professional – patient trusting relationship. In our cohort there was only 34 incidents of misadventure codes (<0.004% of all TEs), and they make no substantive difference to the results.

**S: Note 2 Methods:** exponentiated survival models and time dependent variables

In this paper we use longitudinal survival data, within a generalised linear modelling (GLM) framework. Using Poisson regression, we model incidence rates for non-affective psychosis stratified by interpersonal and non-interpersonal trauma counts. We extend this model by splitting the time scale and age into intervals in which it is reasonable to assume hazard rates for NAPD to remain constant but allow it to vary between intervals – this is known as piecewise exponential modelling. We start with one row of data for each observation (participant) we then split the time scale into calendar time and age intervals (we have used a 5-year intervals) –we then have more than one row of each subject (pseudo-observations). Although we have multiple rows of data per subject there is no need to be concerned with correlation between observations on the same subject, as the time intervals are non-overlapping so the conditional probability contribution to the likelihood product will equal the maximum likelihood.

We enter the trauma exposure variable (administrative service contact for trauma related diagnoses) at the time-of-service contact. This is again achieved by using episode splitting. The data set is further split - before and after the trauma which indicates the change from 0🡪 1 count,1🡪2 counts etc. This ensures a better measure of person time exposure. This means that the trauma exposures examined are all prior to the first diagnosis of NAPD this is possible as we know the date of all contact to services, from the administrative registers and trauma experiences were entered into the model as a time dependent variable - we followed the cohort up from the age of 16 – as they entered the period of increased risk for non- affective psychosis, until December 31^st^ 2016 when the study ended. The trauma exposure at age 16, is the count of administratively coded trauma contacts for interpersonal traumatic events and non-interpersonal traumatic events - before age 16. After age 16 any administratively defined trauma (either interpersonal or non-interpersonal) is then entered into the survival framework at the specific point in time the person contacts services with a predefined trauma related diagnostic code and this episode is then added to the ‘running count’. This is done by episode splitting- so that every time the trauma count changes, the data set is split at that point in time.

The inter-personal and non-interpersonal trauma count 0,1,2,3+ was included in the model as an indicator variable. We also estimated a rate ratio linear trend by entering the explanatory variable as a continuous 0,1,2,3.

In the mediation analysis a simple dichotomised variable of any inter-personal traumatic events was used as the statistical methods used are new and have been shown to perform better using dichotomised exposure.

**S: Table 1: Cohort characteristics by non-affective psychosis status**

|  | Outcome status (Non-affective Psychosis) | |  |
| --- | --- | --- | --- |
|  | No | Yes | Total |
| Participants (row %) | 1,855,940 (99.06) | 17,653 (0.94) | 1,873,593 |
| Females (%) | 903,436 (48.68) | 7,044 (39.90) | 910,480 (48.60) |
| Second generation Migrant (%) | 270,527 (14.58) | 4,266 (24.17) | 274,793 (14.67) |
| Father > = 40 at participant birth (%) | 121,260 (6.63) | 1,525 (8.64) | 122,785 (6.55) |
| Lowest educational attainment (%) | 158,986 (8.57) | 5,354 (30.33) | 164,340 (8.77) |
| Parents in lowest SES at birth (%) | 308,204 (16.61) | 3,895 (22.06) | 312,099 (16.66) |
| Family History NAPD (%) | 38,771 (2.09) | 1,672 (9.47) | 40,443 (2.16) |
| Family History Bipolar Disorder (%) | 36,579 (1.97) | 997 (5.65) | 37,576 (2.01) |
| Family History of Depression (%) | 227,391 (12.25) | 3,975 (22.52) | 231,366 (12.35) |
| No traumatic events (%) | 1,544,878 (83.24) | 14,618 (82.81) | 1,559,496 (83.24) |
| 1 or more non-interpersonal TE (%) | 296,900 (16.00) | 2,669 (15.12) | 299,569 (15.99) |
| 1 or more interpersonal TE (%) | 14,162 (0.77) | 366 (2.08) | 14,528 (0.78) |
| PTSD diagnosis | 10,140 (0.55) | 1,085 (0.61) | 11,225(0.60) |

**S Table 1b : The number of non-interpersonal and interpersonal traumatic experiences entered in the survival analyses with NAPD as an outcome.**

Non-interpersonal and Interpersonal traumatic experiences were entered into the model as time-varying variables. This means we have a value for the variable at t=0, and at least once at t>0 which is the time in the follow up period when a further TE was recorded in the National Patient Register. For example, a subject has no TE exposure at t=0. After one year of follow up they experience a serious RTA which is coded in the National Patient Register and from this point they will contribute person time to exposure of 1 non-interpersonal TE from this time point. If 3 years after this, they experience the death of a child then similarly when the date the death was registered is the time they would start to contribute person time to 2 TE exposure and so on.

| **Number of Traumatic Events included in the Poisson model when NAPD is the outcome of interest and TEs as the explanatory variable** | | |
| --- | --- | --- |
| Type of TE | # entries | % total population* |
| 1 **non-**interpersonal TE | 292,612 | 14.96% |
| 2 **non**-interpersonal TE | 33,615 | 1.72% |
| 3 **non**-interpersonal TE | 4,578 | 0.23% |
|  | | |
| 1 interpersonal TE | 14,784 | 0.76% |
| 2 interpersonal TE | 1,589 | 0.08% |
| 3 interpersonal TE 1 | 359 | 0.02% |

*1,873,593

**S Table 2: Time-lagged association estimates between TE and NAPD**

| **Traumatic Events** | **Fully adjusted model** ^b^ | | | |
| --- | --- | --- | --- | --- |
|  | **Rate Ratio**  **linear trend** | **P Value** | **95% C.I.** | |
| Non-interpersonal TE |  | | | |
| **No time-lag** | **1.27** | **<0.0001** | **1.23** | **1.31** |
| 1-year time-lag | 1.25 | <0.0001 | 1.20 | 1.29 |
| 2-year time-lag | 1.25 | <0.0001 | 1.20 | 1.29 |
| 3-year time-lag | 1.23 | <0.0001 | 1.19 | 1.28 |
| 4-year time lag | 1.22 | <0.0001 | 1.17 | 1.27 |
| 5-year time lag | 1.21 | <0.0001 | 1.16 | 1.27 |
| Interpersonal TE |  | | | |
| **No time lag** | **2.17** | **<0.0001** | **2.02** | **2.33** |
| 1-year time lag | 2.14 | <0.0001 | 1.98 | 2.24 |
| 2-year time lag | 2.05 | <0.0001 | 1.89 | 2.24 |
| 3-year time-lag | 1.98 | <0.0001 | 1.81 | 2.19 |
| 4-year time-lag | 1.96 | <0.0001 | 1.76 | 2.19 |
| 5-year time lag | 1.95 | <0.0001 | 1.75 | 2.16 |

^b^ Full Model: adjusted for age, sex (& their interaction), calendar period, paternal lifetime history of NAPD,

Depressive disorders and Bipolar-Disorder, socioeconomic status, paternal. age at conception, compulsory

school education attainment, second generation migrant status

Here we present the rate ratio linear trends for purely computational efficiency.

**S: Table 3: Association of adulthood TE with NAPD**

| **Traumatic Events** | Full model unadjusted for childhood TE exposure | | | | Full Model **adjusted**  for childhood TE | | | | Full Model **adjusted** for  childhood TE Lagged IRR (5-yrs) | | | |
| --- | --- | --- | --- | --- | --- | --- | --- | --- | --- | --- | --- | --- |
| **Number of Non-interpersonal TE** | **IRR** | **p-value** | **95% C.I.** | | **IRR** | **p-value** | **95% C.I.** | | **IRR** | **p-value** | **95% C.I.** | |
| 1 | 1.42 | <0.0001 | 1.34 | 1.50 | 1.25 | <0.0001 | 1.18 | 1.32 | 1.2 | <0.0001 | 1.13 | 1.28 |
| 2 | 2.26 | <0.0001 | 1.94 | 2.63 | 1.81 | <0.0001 | 1.56 | 2.11 | 1.56 | <0.0001 | 1.28 | 1.89 |
| 3 or more | 2.49 | <0.0001 | 1.67 | 3.72 | 1.79 | 0.004 | 1.2 | 2.68 | 1.22 | 0.512 | 0.67 | 2.2 |
| **Rate Ratio Linear Trend** | **1.43** | **<0.0001** | **1.37** | **1.49** | **1.27** | **<0.0001** | **1.21** | **1.32** | **1.2** | **<0.0001** | **1.14** | **1.27** |
| **Number of interpersonal TE** |  | | | | | | | | | | | |
| 1 | 3.83 | <0.0001 | 3.43 | 4.27 | 2.62 | <0.0001 | 2.34 | 2.93 | 2.13 | <0.0001 | 1.85 | 2.45 |
| 2 | 6.47 | <0.0001 | 4.87 | 8.60 | 4.08 | <0.0001 | 3.07 | 5.43 | 3.75 | <0.0001 | 2.63 | 5.34 |
| 3 or more | 9.80 | <0.0001 | 6.25 | 15.4 | 5.8 | <0.0001 | 3.7 | 9.11 | 5.46 | <0.0001 | 3.16 | 9.8 |
| **Rate Ratio Linear Trend** | **2.78** | **<0.0001** | **2.60** | **2.98** | **2.16** | **<0.0001** | **2.01** | **2.32** | **2.00** | **<0.0001** | **1.79** | **2.16** |

^b^ Full Model: adjusted for age, sex (& their interaction), calendar period, paternal lifetime history of NAPD, Depressive disorders and Bipolar-Disorder, socioeconomic status, paternal. age at conception, compulsory school education attainment, second generation migrant status

**S: Table 4: Association between TE and**

**Acute Stress Reaction (ASR)**

| **Outcome: Acute Stress Reaction** | | | | | | | | |
| --- | --- | --- | --- | --- | --- | --- | --- | --- |
| Traumatic Events | **Model 1(forced)**  adjusted for age, sex & their interaction, calendar year | | | | **Model 2 (full)**  Adjusted forced + complement of confounder variables ^b^ | | | |
|  | **IRR** | **P Value** | **95% C.I.** | | **IRR** | **P Value** | **95% C.I.** | |
| # Non-interpersonal TE |  | | | |  |  |  |  |
| 1 | 1.53 | < 0.0001 | 1.49 | 1.58 | 1.53 | <0.0001 | 1.49 | 1.57 |
| 2 | 2.22 | <0.0001 | 2.07 | 2.39 | 2.22 | <0.0001 | 2.07 | 2.39 |
| 3 or more | 3.32 | <0.0001 | 2.81 | 3.91 | 2.80 | <0.0001 | 2.80 | 3.89 |
| **Rate Ratio Linear Trend** | **1.54** | **<0.0001** | **1.51** | **1.57** | **1.51** | **<0.0001** | **1.47** | **1.54** |
| # Interpersonal TE |  |  |  |  |  |  |  |  |
| 1 | 4.42 | <0.0001 | 4.10 | 4.76 | 4.39 | <0.0001 | 4.07 | 4.74 |
| 2 | 7.49 | <0.0001 | 6.04 | 9.30 | 7.40 | <0.0001 | 5.96 | 9.18 |
| 3 or more | 7.35 | <0.0001 | 4.84 | 11.17 | 7.25 | <0.0001 | 4.77 | 11.01 |
| **Rate Ratio Linear Trend** | **3.01** | **<0.0001** | **2.87** | **3.17** | **3.00** | **<0.0001** | **2.85** | **3.15** |

^b^ Full Model: adjusted for age, sex (& their interaction), calendar period, paternal lifetime history of NAPD, Depressive disorders and Bipolar-Disorder, socioeconomic status, paternal. age at conception, compulsory school education attainment, second generation migrant status
